# Real-world glycemic outcomes of a tubeless automated insulin delivery system: a single-center observational study in Italy

**DOI:** 10.3389/fendo.2025.1717249

**Published:** 2025-12-15

**Authors:** Silvia Angelino, Maria Ida Maiorino, Michela Petrizzo, Nicole Di Martino, Paola Caruso, Carla Carbone, Alessandro Pontillo, Antonietta Maio, Miriam Longo, Lorenzo Scappaticcio, Giuseppe Bellastella, Katherine Esposito

**Affiliations:** 1 Department of Advanced Medical and Surgical Sciences, University of Campania Luigi Vanvitelli, Naples, Italy; 2 Unit of Endocrinology and Metabolic Diseases, University Hospital Luigi Vanvitelli, Naples, Italy; 3 PhD Program in Translational Medicine, University of Campania Luigi Vanvitelli, Naples, Italy; 4 Department of Life Science, Health, and Health Professions, Link Campus University, Roma, Italy

**Keywords:** continuous glucose monitoring, continuous subcutaneous insulin infusion, Omnipod 5, patch pump, time in range, type 1 diabetes

## Abstract

**Background:**

The objective of this study is to describe the short-term change observed in CGM-related measures and relevant clinical variables in individuals with type 1 diabetes transitioning to Omnipod 5 insulin treatment within a real-world setting.

**Methods:**

The study involved adults with type 1 diabetes treated with Omnipod 5, whose data were collected over a 14-days observation period prior to (Time 0) and following the (Time 1) initiation of the patch pump use.

**Results:**

A total of 20 adults with well-controlled glycemia were included in the study. From baseline to follow-up, Time in Range (TIR) significantly increased from 57.3% to 67.3% (P<0.001). Concurrently, there were significant decreases in Time Above Range (TAR) Level 1 (mean difference, -4.7 ± 6.1%, P = 0.003) and Level 2 (-4.2 ± 6.1%, P = 0.018), as well as in Time Below Range (TBR) Level 1 (-1.0 ± 1.1%, P<001), TBR Level 2 (-0.4 ± 0.5%, P = 0.015), and Glycemia Risk Index (-13.8 ± 15.1 P<0.001). Importantly, no significant changes in insulin doses were observed during the study period.

**Conclusions:**

Omnipod 5 initiation allowed participants to improve CGM-related metrics and the quality of glucose control in the short-term, without increasing the need for insulin.

## 1 Introduction

Diabetes care has been revolutionized by advances in glucose sensors and insulin pump technology, with recent standards of care emphasizing their early use in the management of people on insulin therapy (1, 2). However, the achievement of glucose control remains challenging in type 1 diabetes, representing a high burden of disease that requires a high intensity of care (3–5). Over the last decade, a notable improvement in glycemic outcomes was observed in people with type 1 diabetes, concurrent with the increased uptake of diabetes technology (6–11).

Automated insulin delivery (AID) systems have been shown to improve glycemic outcomes in people with type 1 diabetes, by lowering hemoglobin A1c (HbA1c), increasing time in range (TIR) and reducing mean glucose and time in hyperglycemia derived from continuous glucose monitoring (CGM) (12–16). Most of the available AID systems consist of tethered pumps which deliver insulin to the infusion site cannula through a length of tubing; only few existing AID systems use a tubeless insulin pump (17, 18). These have been developed to reduce important sources of error related to cannula injection site (occlusions, tubing kinks and others), and to improve the portability, since they are smaller, lighter and allow greater freedom of movement than conventional pumps (19).

Among these systems, Omnipod 5 is one of the newest hybrid closed loop. Compared with the other AID systems currently available, Omnipod 5 exhibits some distinctive design and functional features. It operates through a tubeless, wearable Pod that integrates both the insulin reservoir and the automated control algorithm. This configuration allows for continuous insulin delivery even in absence of active interaction between the pump and the controller/smartphone, improving comfort and favoring discretion for users. Some attractive functional features of this system include the algorithm predicting glucose level within 60 minutes, the every-5 minutes modifiable insulin administration, and the customizable glycemic target (18, 20). The safety and efficacy of Omnipod 5 system have been investigated in clinical studies of both adults and children with type 1 diabetes, demonstrating a significant improvement of HbA1c levels and TIR, with a low incidence of hypoglycemia (20–22). Moreover, the use of Omnipod 5 was associated with benefit in terms of diabetes-related psychosocial outcomes in people with type 1 diabetes and their caregivers (23, 24).

The Omnipod 5 AID System was commercially launched in Italy for individuals with type 1 diabetes on January 15, 2025. However, the Diabetes Unit of the University Hospital of Campania Luigi Vanvitelli, representing a tertiary care center in Naples, received early authorization to prescribe the system starting in December 2024, so that people followed at the outpatient clinic of the Diabetes Center had the opportunity to discover and practice with Omnipod 5, prior to his national release. This represents the setting to conduct an observational study on the initial cohort of Italian Omnipod 5 users. In this context, we therefore aim to describe the early changes in the short term CGM-related metrics and additional relevant clinical measures in people with type 1 diabetes initiating insulin treatment with Omnipod 5 in a real-world setting.

## 2 Materials and methods

### 2.1 Study design and population

The present study was carried out according to the Strengthening the Reporting of Observational Studies in Epidemiology (STROBE) Statement (25). The STROBE checklist is reported in the **Supplementary Material**.

This is a single center, observational prospective study that involved adults with type 1 diabetes followed at Diabetes Unit of the University Hospital of Campania “Luigi Vanvitelli” in Naples (Italy), from December 2024 to February 2025. We included men and women, aged >18 years, diagnosed with type 1 diabetes, with HbA1c <10%, either technology-naïve and treated with insulin pump and/or CGM, who were initiated to Omnipod 5 (Insulet Corporation, Acton, MA) and were using it in Automated Mode with the same pod settings (glycemic target equal to 120 mg/dl and duration of insulin action of three hours). The exclusion criteria were 1) presence of acute diabetes-related disorders, 2) pregnancy and/or breastfeeding, 3) acute illness or concomitant chronic disease that may interfere with glucose control, 4) intolerance or allergy to the adhesive tape or any unresolved skin condition in the area of sensor or pump placement, 5) percentage of CGM sensor and patch pump use < 70% during the time of observation, 6) different pod settings, including glycemic target and duration of insulin action, that are able to influence its functioning.

### 2.2 Institutional Review Broad (IRB) approval statement

The study was approved by the Medical Ethics Committee of University of Campania “Luigi Vanvitelli” (protocol number 02_1012_2024) and complies with the Declaration of Helsinki and the International Conference on Harmonization/Good Clinical Practice Guidelines. All participants signed an informed consent before enrollment.

### 2.3 Clinical variables and definition of periods

Demographic and clinical variables were collected at baseline, and included age, sex, age at diagnosis and duration of diabetes, body weight, body mass index (BMI), waist circumference, systolic (SBP) and diastolic blood pressure (DBP), HbA1c, fasting plasma glucose (FPG), lipid profile (total, HDL, and LDL cholesterol, and triglycerides), renal function based on creatinine levels and estimated glomerular filtration rate (eGFR, calculated with EPI-CKD formula) (26), previous therapy [multiple daily insulin injections (MDI), continuous subcutaneous insulin infusion (CSII), continuous glucose monitoring (CGM), self-blood glucose monitoring (SBGM)], presence of micro and macrovascular complications, and diagnosis of other concomitant autoimmune diseases.

For each participant, CGM-related metrics were extracted from the dedicated web-based platform (Glooko ^®^) associated with Omnipod 5. Data were collected over a 14-day observation period immediately prior to (Time 0, representing standard therapy) and following (Time 1) the initiation of Omnipod 5 use. The metrics included time in range (TIR, 70–180 mg/dl), tight time in range (70–140 mg/dl), time above range (TAR) level 1 (180–250 mg/dl) and level 2 (>250 mg/dl), time below range (TBR) level 1 (70–55 mg/dl) and level 2 (<55 mg/dl), the coefficient of variation (CV), the glucose monitoring indicator (GMI), the mean glucose and standard deviation. We also calculated the Glycemia Risk Index (GRI), a new metric of glucose control quality, by using the formula “(3.0 × TBR level 2) + (2.4 × TBR level 1) + (1.6 × TAR level 2) + (0.8 × TAR level 1)” (27). Insulin doses (including basal, bolus, total insulin doses and daily insulin doses per kg) were collected from medical records and web-based platform.

### 2.4 Statistical analysis

The primary outcome was the mean difference in TIR from Time 0 to Time 1. Secondary outcomes included the mean difference of other CGM-related metrics, namely TBR (level 1 and level 2), TAR (level 1 and level 2), CV, GMI, mean glucose and standard deviation, GRI, and daily insulin doses. A sample size calculation based on primary outcome was performed, assuming a mean difference of 15.6% and a SD of 11.5% (20). Given an α equal to 0.05 and a β equal to 0.80, a sample size of 14 participants was calculated (28).

Descriptive statistics were used to characterize the study sample. The Kolmogorov-Smirnov test was used to assess whether the variables had a normal distribution. Continuous variables were summarized using mean and standard deviation (SD). If data needed to be presented as median and interquartile range (IQRs), as for sample distribution, the corresponding mean and SD were estimated using the statistical formula described by Wan et al. (29). Categorical variables were represented with frequencies and percentages. Paired t-test or Signed Rank Test were performed to evaluate change of each variable from Time 0 to Time 1, as appropriate. The results were described in the overall population and in subgroups of participants, defined according to the previous insulin regimen (CSII-naïve and CSII-experienced), the sex (male and female), the baseline HbA1c value (HbA1c≥7% or HbA1c<7%), the BMI [normal weight (BMI between 18.5 and 24.9 kg/m^2^) and overweight/obesity (BMI≥25 kg/m^2^)], and the diabetes duration (equal/higher or lower than 15 years). T-test or Rank Sum test were performed to describe the difference of CGM-related metrics between the aforementioned subgroups, as required. Homogeneity of variances was assessed using Levene’s test. Statistical significance was accepted at P<0.05.

## 3 Results

Thirty-three adults with type 1 diabetes treated with Omnipod 5 were screened for the inclusion in the study. After excluding thirteen subjects according to exclusion criteria, twenty were included in the study.

The baseline clinical characteristics of the study population are listed in **Table 1**. A total of 20 adults, with half of them being female (n=10), a mean age of 28.2 years and a mean diabetes duration of 19.2 years, were included in the study. The mean BMI value was 23.3 kg/m^2^, and the mean value of HbA1c was 7.1%. All participants were previously CGM-users, and fourteen individuals have already experienced CSII therapy (twelve of these have been treated with sensor augmented pump). Microvascular diabetes-related complications were recorded in two subjects and were represented by non-proliferative diabetic retinopathy, while no macrovascular complications were reported. Half of the population had diagnosis of other autoimmune diseases.

**Table 1:** Clinical and demographic characteristics of participants in the study.

Variables	Entire study cohort (n=20)
Age, years	28.2 ± 7.2
Diabetes duration, years	19.2 ± 11.1
Female, n (%)	10 (50)
Body weight, kg	68.2 ± 11.7
BMI, kg/m^2^	23.3 ± 4.1
WC, cm	85.6 ± 3.8
Overweight, n (%)	8 (40.0)
SBP, mmHg	117.5 ± 10.0
DBP, mmHg	76.7 ± 8.0
Fasting plasma glucose, mg/dl	158.0 ± 43.9
HbA_1c_, %	7.1 ± 1.1
Total Cholesterol, mg/dl	153.1 ± 43.9
HDL Cholesterol, mg/dl	56.3 ± 19.9
LDL Cholesterol, mg/dl	90.8 ± 17.2
Triglycerides, mg/dl	53.5 ± 18.4
Creatinine, mg/dl	0.8 ± 0.1
eGFR, ml/min/1,73 m^2^	115.9 ± 12.5
CSII users, n (%)	14 (70.0)
Total daily insulin dose, UI/die	46.1 ± 15.8
Basal daily insulin dose, UI/die	23.2 ± 9.3
Bolus daily insulin dose, UI/die	23.1 ± 7.5
Total daily insulin dose, UI/kg/die	0.7 ± 0.2
Other autoimmune diseases, n (%)	10 (50.0)
Microvascular Complications, n (%)	2 (10.0)

BMI, body mass index; CGM, continuous glucose monitoring; CSII, continuous subcutaneous insulin infusion; DBP, diastolic blood pressure; eGFR, estimated Glomerular Filtration Rate; HbA_1c_, Hemoglobin A1c; HDL cholesterol, High-Density Lipoprotein cholesterol; LDL cholesterol, Low-Density Lipoprotein cholesterol; SBP, systolic blood pressure; WC, waist circumference

**Figure 1:**
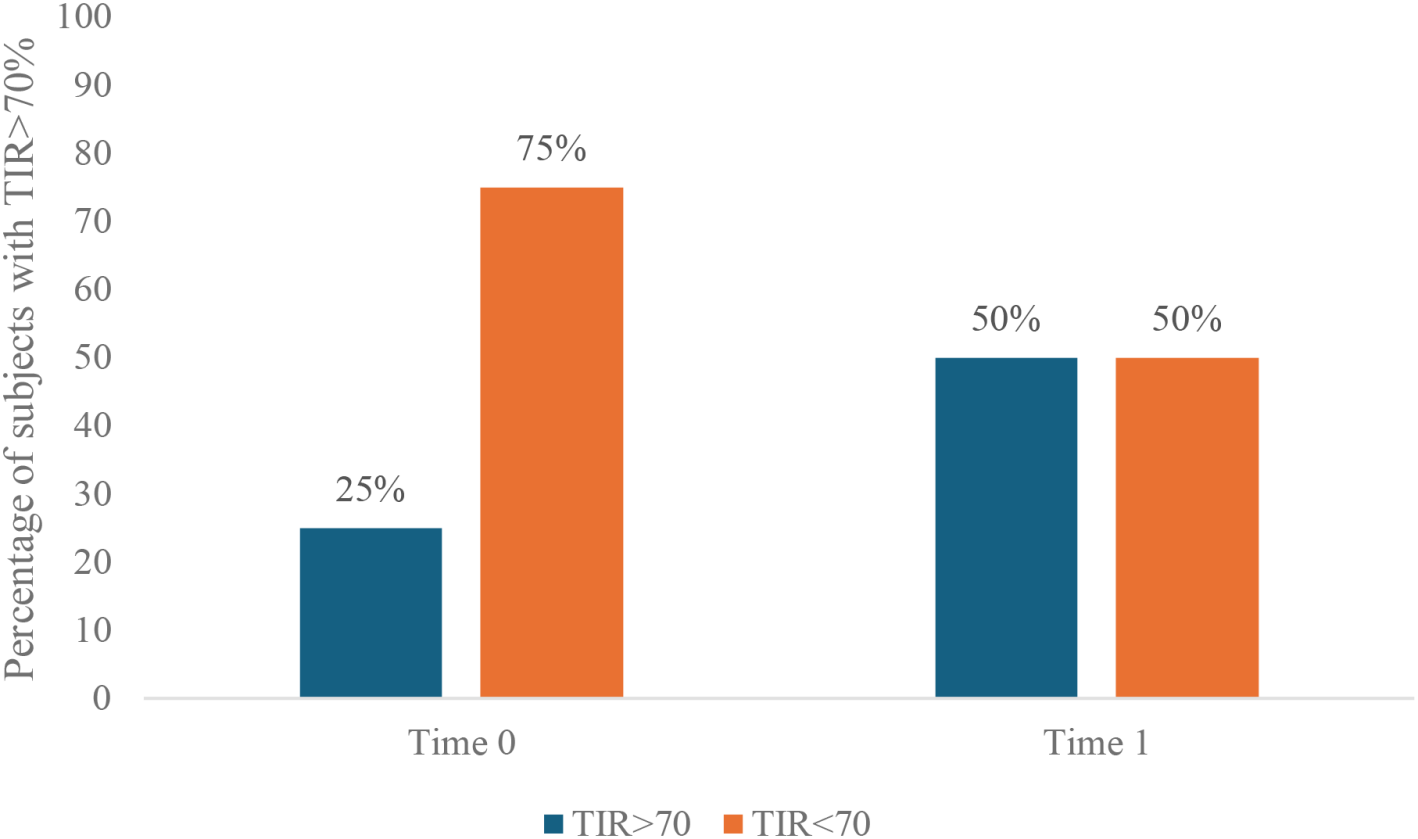
Percentage of subjects with TIR >70% at Time 0 and Time 1 (P = 0.191). TIR, Time In Range.

**Table 2** depicts the variations of CGM-related metrics and total daily insulin doses from Time 0 to Time 1. There was a 10.0% increase in TIR (Time 0 to Time 1, 57.3 ± 16.2% to 67.3 ± 8.3%, P<0.001), associated with a non-significant increase in tight time in range. The percentage of people presenting with TIR>70% doubled, although this change was not statistically significant (**Figure 1**). As for secondary outcomes, there was a significant reduction in TAR level 1 (26.8 ± 6.7% to 22.1 ± 3.9%, P = 0.003), TAR level 2 (13.4 ± 11.4% to 9.2 ± 7.2%, P = 0.018), as well as TBR level 1 (2.1 ± 1.4% to 1.1 ± 0.7%, P<0.001) and TBR level 2 (0.8 ± 0.9% to 0.4 ± 0.7%, P = 0.015); moreover, there was a significant 13.8 improvement of GRI (50.0 ± 22.1 to 36.2 ± 12.3, P<0.001); total daily insulins doses decreased slightly, although this was not statistically significant (**Table 2**).

**Table 2:** Change in CGM-related metrics from Time 0 to Time 1.

Variables	Time 0	Time 1	Difference	P
Time in range, %	57.3 ± 16.2	67.3 ± 8.3	10.0 ± 11.5	<0.001^1^
Tight time in range, %	37.5 ± 10.8	42.0 ± 8.6	4.5 ± 10.9	0.079^1^
TAR level 1, %	26.8 ± 6.7	22.1 ± 3.9	-4.7 ± 6.1	0.003^1^
TAR level 2, %	13.4 ± 11.4	9.2 ± 7.2	-4.2 ± 7.3	0.018^2^
TBR level 1, %	2.1 ± 1.4	1.1 ± 0.7	-1.0 ± 1.1	<0.001^2^
TBR level 2, %	0.8 ± 0.9	0.4 ± 0.7	-0.4 ± 0.5	0.015^2^
CV, %	36.6 ± 4.0	34.4 ± 5.0	-2.2 ± 5.0	0.067^1^
GMI, %	7.4 ± 0.7	7.2 ± 0.3	-0.2 ± 0.5	0.121^2^
Mean glucose, mg/dl	172.3 ± 28.2	163.3 ± 14.0	-9.0 ± 19.9	0.058^2^
SD, mg/dl	60.7 ± 12.3	56.5 ± 11.4	-4.2 ± 8.8	0.047^1^
GRI	50.0 ± 22.1	36.2 ± 12.3	-13.8 ± 15.1	<0.001^2^
Total daily insulin dose, UI/die	46.1 ± 15.8	44.9 ± 13.7	-1.2 ± 9.8	0.587^1^
Basal daily insulin dose, UI/die	23.1 ± 9.3	23.2 ± 7.5	0.1 ± 4.4	0.908^1^
Bolus daily insulin dose, UI/die	23.3 ± 7.6	21.0 ± 9.2	-2.3 ± 6.9	0.151^1^
Insulin dose per kg, UI/kg/die	0.7 ± 0.2	0.7 ± 0.2	0.0 ± 0.1	0.929^1^

All data are expressed as mean ± SD.

CV, coefficient of variation; GMI, Glucose Monitoring Indicator; GRI, Glycemia Risk Index; SD, standard deviation; TAR, Time Above Range; TBR, Time Below Raange.

^1^Paired t-test.

^2^Wilcoxon signed rank test.

**Tables 3**, **4** show the subgroup analyses of the change of TIR and GRI, respectively. A significant difference in the change of TIR was observed in favor of people with previous CSII therapy compared to those with previous MDI therapy (P = 0.033) and in participants with baseline HbA1c ≥7% than in those with HbA1c <7% (P = 0.027) (**Table 3**). On the other hand, GRI variations were larger in males than in females (P = 0.031), in subjects with baseline HbA1c ≥7% than those with baseline HbA1c <7% (P = 0.012), and in people who had previously received CSII therapy than in those with previous use of MDI insulin regimen (P = 0.048).

**Table 3:** Subgroup analysis of the TIR change in the study population.

Subgroup	Sample (n)	Mean ± DS	95%CI	P^1^	P^2^
Sex					0.088
Male	10	13.3 ± 10.0	6.1, 20.5	0.002	
Female	10	6.7 ± 12.4	-2.2, 15.6	0.122	
BMI					0.939
<25 Kg/m^2^	12	9.8 ± 8.9	4.2, 15.5	0.003	
≥25 Kg/m^2^	8	10.3 ± 15.3	-2.5, 23.0	0.100	
HbA1c					0.027
<7%	11	4.8 ± 8.7	-1.0, 10.6	0.094	
≥ 7%	9	16.3 ± 11.8	7.3, 25.4	0.003	
Diabetes duration					0.177
<15 years	7	6.1 ± 10.7	-3.8, 16.1	0.181	
≥15 years	13	12.1 ± 11.7	5.0, 19.2	0.003	
Previous insulin regimen					0.033
CSII-naive	6	1.8 ± 4.5	-2.9, 6.6	0.368	
CSII-experienced	14	13.5 ± 11.9	6.6, 20.4	<0.001	

All data are expressed as mean ± SD.

BMI, body mass index; CSII, continuous subcutaneous insulin infusion.

^1^Paired sample t-test.

^2^Indipendent sample t-test.

**Table 4:** Subgroup analysis of the GRI change in the study population.

Subgroup	Sample (n)	Mean ± DS	95%CI	P^1^	P^2^
Sex					0.031
Male	10	-19.0 ± 11.9	-27,5, -10,5	<0.001	
Female	10	-8.6 ± 16.6	-3.3, 20.5	0.137	
BMI					0.813
<25 kg/m^2^	12	-13.1 ± 11.1	-20.2, -6.1	0.002	
≥25 kg/m^2^	8	-14.8 ± 20.5	-31.9, 2.3	0.080	
HbA1c					0.012
<7%	11	-6.6 ± 10.8	-0.6, -13.9	0.068	
≥ 7%	9	-22.6 ± 15.4	-34.4, -10.8	0.002	
Diabetes duration					0.153
<15 years	7	-8.7 ± 14.2	-21.9, 4.4	0.155	
≥15 years	13	-16.5 ±1 5.3	-25.8, -7.3	0.002	
Previous insulin regimen					0.048
CSII-naive	6	-3.8 ± 7.3	-11.5, 3.9	0.259	
CSII-experienced	14	-18.1 ± 15.6	-27.1, -9.1	<0.001	

All data are expressed as mean ± SD.

BMI, body mass index; CSII, continuous subcutaneous insulin infusion.

^1^Paired sample t-test.

^2^Indipendent sample t-test.

The effects of Omnipod 5 exposure in patient subgroups are detailed in the subgroup analyses provided within the supplemental material (**Supplementary Tables 1–10**). The most favorable outcomes, specifically regarding increased TIR, reduced TAR and TBR, and lower GRI were observed in study participants previously treated with insulin pumps, worse baseline glycemic control (HbA1c ≥ 7.0%), lower BMI, longer duration of diabetes (>15 years). Furthermore, a significant gender difference was noted, with males achieving superior results compared to females. Variations of CGM-related metrics were mostly registered in absence of significant changes in insulin doses, with the exception of participants with BMI ≥25 kg/m^2^, who reported a reduction of daily insulin doses.

## 4 Discussion

This study reports the inaugural Italian experience with the Omnipod 5 AID system in adults with type 1 diabetes within a real-world clinical setting. We found clinically significant improvements across most metrics of glycemic control from 14 days pre- to 14 days post-device initiation.

Our cohort comprised adults with suboptimal glyco-metabolic control who were generally free from diabetes-related complications, whose indication to use an AID system was driven by the improvement of the overall glucose control. Indeed, at baseline, most participants were outside target ranges for most CGM-metrics, with the exception of TBR, as defined by the International Consensus on time in range (3). More than half of the study population had prior experience with insulin pump therapy before starting the treatment with Omnipod 5. Participants underwent improvement in glycemic control, highlighted by expanded time spent in normoglycemia, with less hyperglycemia and hypoglycemia. Moreover, we recorded a significant improvement in GRI, a comprehensive single-number summary of the quality of glucose control, weighted by the combined risk of hypoglycemia and hyperglycemia; lower GRI values indicate optimal glucose control (27). The percentage of people achieving the recommended target for TIR doubled from baseline to follow-up, although this increase did not reach statistical significance, probably due to the small sample size (3). Importantly, the clinically remarkable gains in glucose control occurred without the need to increase total daily insulin doses.

These findings are consistent with results from trials evaluating the safety and effectiveness of Omnipod 5 system in larger cohorts with a broad range of age (20–22, 30, 31). Notably, the 3-month pivotal trial by Brown et al. demonstrated a significant improvement in the time in target range for both children and adults (by 15.6% and 9.3%, respectively) associated with a low incidence of hypoglycemia and the reduction in HbA1c (20). Subsequent trials with extended follow-up and recent observational studies have further corroborated these findings (21, 22, 30–33). Moreover, a recent comparative analysis further supported the clinical advantages and unique usability profile of Omnipod 5 within the broader AID landscape in reducing hypoglycemia and ameliorating glucose control during physical exercise (34).

A crucial aspect of our study is that the glucose target in Omnipod 5 was uniformly set at 120 mg/dl for all participants. The customizable glucose target setting is a critical parameter for AID systems, as it directly impacts the algorithm performance (22). We could speculate that a more ambitious glucose target 110 mg/dl might have yielded additional benefits to glycemic control in our participants, consistent with findings reported by Forlenza et al. (22, 34, 35).

Subgroup analyses of glycemic outcomes revealed consistently successful utilization of Omnipod 5 across all participant groups. Of note, the amelioration of TIR was more pronounced in individuals previously using CSII therapy, compared to those who were CSII-therapy naïve. This observation may be explained by the fact that participants with prior use of MDI therapy exhibited better baseline TIR compared to those on CSII therapy. Nevertheless, it is well-established that transitioning from MDI to AID therapy generally confers substantial benefits in terms of glucose control (4).

Interestingly, participants with a BMI ≥25 kg/m^2^ experienced a reduction in daily insulin doses concurrently with a decrease of time spent in hypoglycemia. This finding supports previous evidence and suggests that Omnipod 5 could contribute to a more appropriate distribution of insulin doses throughout the day, potentially addressing issues related to insulin requirements and weight-promoting effect in overweight and obese people (20, 36).

This study has several strengths, including 1) the selection of a balanced study population with similar proportions of both sexes and individuals previously treated with CSII or MDI therapy, 2) the consistent use of identical Omnipod 5 settings, including the glucose target and the time of insulin activity, that ensured a uniform starting point for the evaluation of glycemic control across the study population, 3) the availability of a significant amount of clinical data collected in a real-world setting. However, the findings should be interpreted in light of several limitations. Foremost among these are the observational design, lacking of a control group, the very small sample size due to the recent introduction of the device in Italy, and the short follow-up period. The observed improvements in glucose control might, in part, be attributed to a “novelty factor” associated with initiating a new system.

In conclusion, adults with type 1 diabetes starting Omnipod 5 use demonstrated a short-term improvement in key CGM-related metrics, without requiring an increase in insulin dosage. The current study confirms the beneficial effects on short-term glucose control provided by new tubeless AID system, that represents a therapeutic option to be adopted early in the routine clinical care. Further research is warranted to support the long-term durability of the system’s effectiveness and its capacity to achieve all the treatment goals, including the potential to reduce the risk of acute and chronic complications. 

## Data Availability

The raw data supporting the conclusions of this article will be made available by the authors, without undue reservation.
