# Supplementary material for: Real-world glycemic outcomes of a tubeless automated insulin delivery system: a single-center observational study in Italy
Source: Front Endocrinol (Lausanne). 2025 Dec 15;16:1717249. doi: 10.3389/fendo.2025.1717249 (PMC12745233; doi:10.3389/fendo.2025.1717249)
Supplement: Supplementary file 1 [file DataSheet1.docx]

**Real-World Glycemic Outcomes of a tubeless Automated Insulin Delivery System: A Single-Center Observational Study in Italy**

Supplementary Material

Supplementary Figure 1..………………………………………………………………………….……..pag. 2

Supplementary Table 1…………………………………………………………………………...……....pag. 3

Supplementary Table 2……………………………………………………………………………...…....pag. 4

Supplementary Table 3……………………………………………………………………………...…....pag. 5

Supplementary Table 4……………………………………………………………………………...…....pag. 6

Supplementary Table 5……………………………………………………………………………...…....pag. 7

Supplementary Table 6……………………………………………………………………………...…....pag. 8

Supplementary Table 7……………………………………………………………………………...…....pag. 9

Supplementary Table 8……………………………………………………………………………...…...pag. 10

Supplementary Table 9……………………………………………………………………………...…...pag. 11

Supplementary Table 10…………………………………………………………………………...….....pag. 12

Supplementary Figure 2………………………………………………………………………………….pag. 13

Supplementary Figure 3………………………………………………………………………………….pag. 14

Supplementary Figure 4………………………………………………………………………………….pag. 15

Supplementary Figure 5………………………………………………………………………………….pag. 16

Supplementary Figure 6………………………………………………………………………………….pag. 17

Strobe Statement…………………………………………………………………………..………..……pag. 18


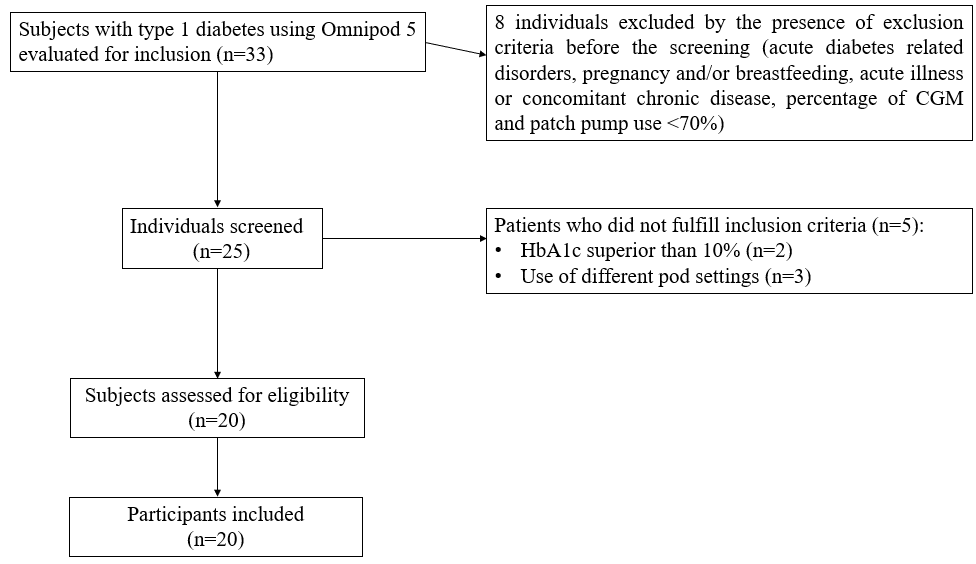


**Supplementary Figure 1 –** Screening flowchart

**Supplementary Table 1**– Change in CGM-related metrics from Time 0 to Time 1 in CSII-naive participants.

| **CSII-naive (n=6)** | | | | |
| --- | --- | --- | --- | --- |
| **Variables** | **Time 0** | **Time 1** | **Difference** | **P** |
| TIR, % | 65.8 ± 8.8 | 67.7 ± 7.8 | 1.9 ± 4.5 | 0.368^1^ |
| TTR, % | 37.7 ± 15.5 | 42.8 ± 6.4 | 5.2 ± 11.2 | 0.311^1^ |
| TAR level 1, % | 22.8 ± 5.3 | 22.0 ± 4.5 | 0.8 ± 3.1 | 0.534^1^ |
| TAR level 2, % | 9.3 ± 4.1 | 9.0 ± 3.5 | 0.3 ± 3.5 | 0.828^1^ |
| TBR level 1, % | 1.8 ± 0.5 | 1.2 ± 0.4 | -0.6 ± 0.5 | 0.025^2^ |
| TBR level 2, % | 0.5 ± 0.5 | 0.2 ± 0.4 | -0.3 ± 0.5 | 0.175^2^ |
| CV, % | 38.1 ± 3.6 | 35.9 ± 2.2 | -2.2 ± 4.7 | 0.309^1^ |
| GMI, % | 7.1 ± 0.3 | 7.2 ± 0.2 | 0.1 ± 0.2 | 0.542^1^ |
| Mean glucose, mg/dl | 161.0 ± 13.0 | 160.7 ± 9.4 | -0.3 ± 7.0 | 0.912^1^ |
| SD, mg/dl | 61.0 ± 8.0 | 57.7 ± 6.3 | -4.3 ± 8.5 | 0.380^2^ |
| GRI | 39.1 ± 11.1 | 35.3 ± 9.7 | -3.8 ± 7.3 | 0.259^1^ |
| Total daily insulin dose, UI/die | 48.2± 14.3 | 45.5 ± 14.9 | -2.7 ± 9.7 | 0.530^1^ |
| Basal daily insulin dose, UI/die | 21.8 ± 7.6 | 21.8 ± 6.5 | 0.0 ± 5.4 | 0.989^1^ |
| Bolus daily insulin dose, UI/die | 26.4 ± 7.1 | 23.2 ± 10.3 | -3.2 ± 6.6 | 0.289^1^ |
| Insulin dose per kg, UI/kg/die | 0.7 ± 0.1 | 0.7 ± 0.2 | -0.0 ± 0.1 | 0.578^1^ |
| All data are expressed as mean ± SD  Abbreviations: CV, coefficient of variation; GMI, Glucose Monitoring Indicator; GRI, Glycemia Risk Index; SD, standard deviation; TAR, Time Above Range; TBR, Time Below Range; TIR, Time In Range; TTR, Tight Time in Range. | | | | |

**Supplementary Table 2** – Change in CGM-related metrics from Time 0 to Time 1 in CSII-experienced participants.

| **CSII-experienced (n=14)** | | | | |
| --- | --- | --- | --- | --- |
| **Variables** | **Time 0** | **Time 1** | **Difference** | **P** |
| TIR, % | 53.6 ± 17.5 | 67.1 ± 8.8 | 13.5 ± 11.9 | <0.001^1^ |
| TTR, % | 37.4 ± 8.9 | 41.7 ± 9.6 | 4.3 ± 11.2 | 0.177^1^ |
| TAR level 1, % | 28.4 ± 6.7 | 22.1 ± 3.8 | -6.4 ± 6.3 | 0.002^1^ |
| TAR level 2, % | 15.1 ± 13.1 | 9.2 ± 8.5 | -5.9 ± 7.9 | 0.016^1^ |
| TBR level 1, % | 2.1 ± 1.6 | 1.1 ± 0.8 | -1.0 ± 1.3 | 0.008^2^ |
| TBR level 2, % | 0.9 ± 1.1 | 0.5 ± 0.9 | -0.4 ± 0.6 | 0.055^2^ |
| CV, % | 36.0 ± 4.2 | 33.8 ± 5.8 | -2.2 ± 5.3 | 0.149^1^ |
| GMI, % | 7.5 ± 0.8 | 7.2 ± 0.4 | -0.3 ± 0.6 | 0.086^1^ |
| Mean glucose, mg/dl | 177.1 ± 31.8 | 164.4 ± 15.7 | -12.7 ± 22.6 | 0.056^1^ |
| SD, mg/dl | 60.6 ± 14.0 | 56.0 ± 13.2 | -4.6 ± 9.3 | 0.088^1^ |
| GRI | 54.7 ± 24.3 | 36.6 ± 13.6 | -18.1 ± 15.6 | <0.001^1^ |
| Total daily insulin dose, UI/die | 45.1 ± 16.9 | 44.6 ± 13.8 | -0.5 ± 10.1 | 0.832^1^ |
| Basal daily insulin dose, UI/die | 23.6 ± 10.2 | 23.8 ± 8.1 | 0.2 ± 4.1 | 0.874^1^ |
| Bolus daily insulin dose, UI/die | 21.9 ± 7.6 | 20.0 ±8.9 | -1.9 ± 7.1 | 0.338^1^ |
| Insulin dose per kg, UI/kg/die | 0.6 ± 0.2 | 0.6 ± 0.1 | 0.0 ± 0.1 | 0.657^1^ |
| All data are expressed as mean ± SD  Abbreviations: CV, coefficient of variation; GMI, Glucose Monitoring Indicator; GRI, Glycemia Risk Index; SD, standard deviation; TAR, Time Above Range; TBR, Time Below Range; TIR, Time In Range; TTR, Tight Time in Range. | | | | |

**Supplementary Table 3 –** Change in CGM-related metrics from Time 0 to Time 1 in female participants.

| **Female (n=10)** | | | | |
| --- | --- | --- | --- | --- |
| **Variables** | **Time 0** | **Time 1** | **Difference** | **P** |
| TIR, % | 58.9 ± 19.6 | 65.5 ± 10.9 | 6.6 ± 12.4 | 0.122^2^ |
| TTR, % | 36.6 ± 12.9 | 42.0 ± 10.0 | 5.4 ± 14.5 | 0.268^1^ |
| TAR level 1, % | 25.6 ± 8.1 | 22.5 ± 4.1 | -3.1 ± 6.0 | 0.136^1^ |
| TAR level 2, % | 13.9 ± 14.3 | 10.8 ± 9.1 | -3.1 ± 9.0 | 0.303^2^ |
| TBR level 1, % | 1.5 ± 0.7 | 0.9 ± 0.6 | -0.6 ± 0.7 | 0.024^2^ |
| TBR level 2, % | 0.2 ± 0.4 | 0.3 ± 0.5 | 0.1 ± 0.3 | 0.343^2^ |
| CV, % | 35.7 ± 4.1 | 34.2 ± 4.8 | -1.5 ± 5.2 | 0.390^2^ |
| GMI, % | 7.4 ± 0.9 | 7.3 ± 0.4 | -0.1 ± 0.6 | 0.632^1^ |
| Mean glucose, mg/dl | 174.2 ± 35.9 | 167.4 ± 18.0 | -6.8 ± 23.2 | 0.378^2^ |
| SD, mg/dl | 57.3 ± 12.8 | 57.7 ± 13.5 | 0.4 ± 7.4 | 0.868^1^ |
| GRI | 46.9 ± 26.3 | 38.3 ± 15.6 | -8.6 ± 16.6 | 0.137^2^ |
| Total daily insulin dose, UI/die | 37.7 ± 14.6 | 34.9 ± 7.2 | -2.8 ± 11.6 | 0.459^1^ |
| Basal daily insulin dose, UI/die | 18.4 ± 7.4 | 18.5 ± 5.1 | 0.1 ± 5.3 | 0.945^1^ |
| Bolus daily insulin dose, UI/die | 19.9 ± 7.1 | 15.5 ± 4.2 | -4.4 ± 7.1 | 0.078^1^ |
| Insulin dose per kg, UI/kg/die | 0.6 ± 0.2 | 0.5 ± 0.1 | -0.1 ± 0.2 | 0.522^1^ |
| All data are expressed as mean ± SD  Abbreviations: CV, coefficient of variation; GMI, Glucose Monitoring Indicator; GRI, Glycemia Risk Index; SD, standard deviation; TAR, Time Above Range; TBR, Time Below Range; TIR, Time In Range; TTR, Tight Time in Range. | | | | |

**Supplementary Table 4 –** Change in CGM-related metrics from Time 0 to Time 2 in male participants.

| **Male (n=10)** | | | | |
| --- | --- | --- | --- | --- |
| **Variables** | **Time 0** | **Time 1** | **Difference** | **P** |
| TIR, % | 55.7 ± 12.7 | 69.0 ± 4.6 | 13.3 ± 10.0 | 0.002^1^ |
| TTR, % | 38.4 ± 8.8 | 42.1 ± 7.4 | 3.7 ± 6.5 | 0.104^1^ |
| TAR level 1, % | 27.9 ± 5.1 | 21.6 ± 3.9 | -6.3 ± 6.0 | 0.009^1^ |
| TAR level 2, % | 12.9 ± 8.2 | 7.6 ± 4.7 | -5.3 ± 5.2 | 0.011^1^ |
| TBR level 1, % | 2.6 ± 1.6 | 1.3 ± 0.8 | -1.3 ± 1.3 | 0.013^1^ |
| TBR level 2, % | 1.3 ± 1.0 | 0.5 ± 0.9 | -0.8 ± 0.4 | <0.001^2^ |
| CV, % | 37.6 ± 3.9 | 34.7 ± 5.5 | -2.9 ± 5.0 | 0.103^1^ |
| GMI, % | 7.4 ± 0.5 | 7.1 ± 0.2 | -0.3 ± 0.4 | 0.050^2^ |
| Mean glucose, mg/dl | 170.4 ± 19.4 | 159.2 ± 7.0 | -11.2 ± 17.0 | 0.067^1^ |
| SD, mg/dl | 64.1 ± 11.3 | 55.3 ± 9.5 | -8.8 ± 8.0 | 0.007^1^ |
| GRI | 53.1 ± 17.9 | 34.1 ± 8.1 | -19.0 ± 11.8 | <0.001^1^ |
| Total daily insulin dose, UI/die | 54.4 ± 12.6 | 54.8 ± 11.2 | 0.4 ± 7.8 | 0.868^1^ |
| Basal daily insulin dose, UI/die | 27.6 ± 8.9 | 27.9 ± 6.8 | 0.3 ± 3.5 | 0.788^1^ |
| Bolus daily insulin dose, UI/die | 26.6 ± 6.6 | 26.5 ± 9.7 | -0.1 ± 6.3 | 0.944^1^ |
| Insulin dose per kg, UI/kg/die | 0.7 ± 0.1 | 0.8 ± 0.1 | 0.1 ± 0.1 | 0.202^1^ |
| All data are expressed as mean ± SD  Abbreviations: CV, coefficient of variation; GMI, Glucose Monitoring Indicator; GRI, Glycemia Risk Index; SD, standard deviation; TAR, Time Above Range; TBR, Time Below Range; TIR, Time In Range; TTR, Tight Time in Range. | | | | |

**Supplementary Table 5 –** Change in CGM-related metrics from Time 0 to Time 1 in participants with baseline HbA1c<7%.

| **HbA1c <7% (n=11)** | | | | |
| --- | --- | --- | --- | --- |
| **Variables** | **Time 0** | **Time 1** | **Difference** | **P** |
| TIR, % | 65.7 ± 11.3 | 70.5 ± 6.7 | 4.8 ± 8.7 | 0.094^1^ |
| TTR, % | 41.7 ± 9.9 | 45.1 ± 8.6 | 3.4 ± 14.1 | 0.448^1^ |
| TAR level 1, % | 24.4 ± 7.1 | 21.2 ± 3.4 | -3.2 ± 5.4 | 0.089^1^ |
| TAR level 2, % | 8.3 ± 5.8 | 7.3 ± 4.2 | -1.0 ± 4.7 | 0.498^1^ |
| TBR level 1, % | 1.5 ± 0.5 | 0.8 ± 0.6 | -0.7 ± 0.8 | 0.012^2^ |
| TBR level 2, % | 0.5 ± 0.5 | 0.2 ± 0.4 | -0.3 ± 0.6 | 0.192^2^ |
| CV, % | 35.4 ± 3.0 | 33.1 ± 4.3 | -2.3 ± 3.0 | 0.031^1^ |
| GMI, % | 7.1 ± 0.4 | 7.1 ± 0.3 | 0.0 ± 0.4 | 0.720^1^ |
| Mean glucose, mg/dl | 159.8 ± 17.7 | 160.0 ± 10.0 | 0.2 ± 13.7 | 0.966^2^ |
| SD, mg/dl | 54.9 ± 11.6 | 53.1 ± 9.0 | -1.8 ± 7.1 | 0.418^1^ |
| GRI | 37.8 ± 13.6 | 31.2 ± 8.3 | -6.6 ± 10.8 | 0.068^2^ |
| Total daily insulin dose, UI/die | 41.4 ± 13.1 | 41.9 ± 15.7 | 0.5 ± 7.4 | 0.816^1^ |
| Basal daily insulin dose, UI/die | 19.7 ± 5.6 | 20.3 ± 6.6 | 0.6 ± 3.5 | 0.625^1^ |
| Bolus daily insulin dose, UI/die | 22.0 ± 7.2 | 20.4 ± 10.4 | -1.6 ± 5.8 | 0.379^1^ |
| Insulin dose per kg, UI/kg/die | 0.6 ± 0.1 | 0.6 ± 0.2 | 0.0 ± 0.1 | 0.799^1^ |
| All data are expressed as mean ± SD  Abbreviations: CV, coefficient of variation; GMI, Glucose Monitoring Indicator; GRI, Glycemia Risk Index; SD, standard deviation; TAR, Time Above Range; TBR, Time Below Range; TIR, Time In Range; TTR, Tight Time in Range. | | | | |

**Supplementary Table 6 –** Change in CGM-related metrics from Time 0 to Time 1 in participants with baseline HbA1c≥7%.

| **HbA1c ≥7% (n=9)** | | | | |
| --- | --- | --- | --- | --- |
| **Variables** | **Time 0** | **Time 1** | **Difference** | **P** |
| TIR, % | 47.0 ± 15.8 | 63.3 ± 8.7 | 16.3 ± 11.8 | 0.003^1^ |
| TTR, % | 32.3 ± 9.9 | 38.3 ± 7.3 | 6.0 ± 5.6 | **0.012** |
| TAR level 1, % | 29.7 ± 5.1 | 23.0 ± 4.5 | -6.7 ± 6.5 | 0.015^1^ |
| TAR level 2, % | 19.7 ± 13.5 | 11.6 ± 9.5 | -8.1 ± 8.1 | 0.017^1^ |
| TBR level 1, % | 2.7 ± 1.8 | 1.4 ± 0.7 | -1.2 ± 1.4 | 0.030^1^ |
| TBR level 2, % | 1.1 ± 1.3 | 0.7 ± 1.0 | -0.4 ± 0.5 | 0.035^2^ |
| CV, % | 38.1 ± 4.8 | 36.1 ± 5.6 | -2.0 ± 6.9 | 0.411^1^ |
| GMI, % | 7.8 ± 0.8 | 7.3 ± 0.4 | -0.5 ± 0.5 | 0.025^1^ |
| Mean glucose, mg/dl | 187.6 ± 31.9 | 167.3 ± 17.5 | -20.3 ± 21.2 | 0.021^1^ |
| SD, mg/dl | 67.8 ± 9.4 | 60.7 ± 13.1 | -7.1 ± 10.2 | 0.071^1^ |
| GRI | 64.9 ± 21.9 | 42.3 ± 14.0 | -22.6 ± 15.4 | 0.002^1^ |
| Total daily insulin dose, UI/die | 51.8 ± 17.7 | 48.4 ± 10.6 | -3.3 ± 12.2 | 0.435^1^ |
| Basal daily insulin dose, UI/die | 27.0 ± 11.4 | 26.8 ± 7.4 | -0.2 ± 5.4 | 0.914^1^ |
| Bolus daily insulin dose, UI/die | 24.8 ± 8.1 | 21.7 ± 8.1 | -3.1 ± 8.2 | 0.289^1^ |
| Insulin dose per kg, UI/kg/die | 0.7 ± 0.2 | 0.7 ± 0.1 | 0.0 ± 0.2 | 0.939^1^ |
| All data are expressed as mean ± SD  Abbreviations: CV, coefficient of variation; GMI, Glucose Monitoring Indicator; GRI, Glycemia Risk Index; SD, standard deviation; TAR, Time Above Range; TBR, Time Below Range; TIR, Time In Range; TTR, Tight Time in Range. | | | | |

**Supplementary Table 7 –** Change in CGM-related metrics from Time 0 to Time 1 in participants with BMI < 25 kg/m^2^.

| **BMI <25 kg/m^2^ (n=12)** | | | | |
| --- | --- | --- | --- | --- |
| **Variables** | **Time 0** | **Time 1** | **Difference** | **P** |
| TIR, % | 58.1 ± 15.4 | 67.9 ± 9.2 | 9.8 ± 8.9 | 0.003^1^ |
| TTR, % | 37.7 ± 8.2 | 43.5 ± 8.4 | 5.8 ± 11.9 | 0.119^1^ |
| TAR level 1, % | 27.0 ± 6.9 | 21.8 ± 3.0 | -5.2 ± 5.9 | 0.011^1^ |
| TAR level 2, % | 12.3 ± 10.6 | 8.8 ± 8.5 | -3.5 ± 5.0 | 0.033^1^ |
| TBR level 1, % | 2.1 ± 1.6 | 1.1 ± 0.8 | -1.0 ± 1.3 | 0.026^2^ |
| TBR level 2, % | 0.8 ± 1.1 | 0.5 ± 0.9 | -0.3 ± 0.6 | 0.104^2^ |
| CV, % | 35.6 ± 3.5 | 33.7 ± 4.7 | -1.9 ± 5.5 | 0.249^1^ |
| GMI, % | 7.4 ± 0.7 | 7.2 ± 0.4 | -0.2 ± 0.4 | 0.111^1^ |
| Mean glucose, mg/dl | 170.3 ± 27.1 | 162.7 ± 16.2 | -7.6 ± 14.9 | 0.103^1^ |
| SD, mg/dl | 58.7 ± 12.1 | 55.1 ± 12.2 | -3.6 ± 10.1 | 0.244^1^ |
| GRI | 48.7 ± 20.3 | 35.6 ± 13.8 | -13.1 ± 11.1 | 0.002^1^ |
| Total daily insulin dose, UI/die | 38.5 ± 13.2 | 42.3 ± 12.3 | 3.8 ± 7.6 | 0.114^1^ |
| Basal daily insulin dose, UI/die | 18.9 ± 6.6 | 21.3 ± 6.1 | 2.4 ± 3.8 | 0.051^1^ |
| Bolus daily insulin dose, UI/die | 20.1 ± 6.7 | 20.2 ± 7.7 | 0.1 ± 6.2 | 0.978^1^ |
| Insulin dose per kg, UI/kg/die | 0.6 ± 0.2 | 0.7 ± 0.2 | 0.1 ± 0.1 | 0.118^1^ |
| All data are expressed as mean ± SD  Abbreviations: CV, coefficient of variation; GMI, Glucose Monitoring Indicator; GRI, Glycemia Risk Index; SD, standard deviation; TAR, Time Above Range; TBR, Time Below Range; TIR, Time In Range; TTR, Tight Time in Range. | | | | |

**Supplementary Table 8 –** Change in CGM-related metrics from Time 0 to Time 1 in participants with BMI ≥25 kg/m^2^

| **BMI ≥25 kg/m^2^ (n=8)** | | | | |
| --- | --- | --- | --- | --- |
| **Variables** | **Time 0** | **Time 1** | **Difference** | **P** |
| TIR, % | 56.1 ± 18.4 | 66.4 ± 7.3 | 10.3 ± 15.3 | 0.100^1^ |
| TTR, % | 37.2 ± 14.5 | 39.8 ± 8.9 | 2.6 ± 9.7 | 0.469^1^ |
| TAR level 1, % | 26.4 ± 6.9 | 22.4 ± 5.2 | -4.0 ± 6.7 | 0.134^2^ |
| TAR level 2, % | 15.1 ± 12.9 | 9.9 ± 5.2 | -5.3 ± 10.1 | 0185^2^ |
| TBR level 1, % | 2.0 ± 1.0 | 1.1 ± 0.6 | -0.9 ± 0.6 | 0.006^2^ |
| TBR level 2, % | 0.6 ± 0.7 | 0.3 ± 0.5 | -0.3 ± 0.5 | 0.080^2^ |
| CV, % | 38.2 ± 4.4 | 35.6 ± 5.5 | -2.6 ± 4.6 | 0.158^1^ |
| GMI, % | 7.5 ± 0.8 | 7.3 ± 0.3 | -0.2 ± 0.7 | 0.463^1^ |
| Mean glucose, mg/dl | 175.3 ± 31.3 | 164.3 ± 10.7 | -11.0 ± 26.8 | 0.284^1^ |
| SD, mg/dl | 63.8 ± 12.8 | 58.6 ± 10.6 | -5.1 ± 7.1 | 0.082^1^ |
| GRI | 52.0 ± 26.0 | 37.2 ± 10.4 | -14.8 ± 20.5 | 0.080^1^ |
| Total daily insulin dose, UI/die | 57.3 ± 12.7 | 48.6 ± 15.7 | -8.7 ± 7.7 | 0.015^1^ |
| Basal daily insulin dose, UI/die | 29.4 ± 9.5 | 26.1 ± 8.9 | -3.3 ± 2.8 | 0.013^1^ |
| Bolus daily insulin dose, UI/die | 27.9 ± 6.4 | 22.1 ± 11.5 | -5.8 ± 6.7 | 0.043^1^ |
| Insulin dose per kg, UI/kg/die | 0.7 ± 0.1 | 0.6 ± 0.1 | 0.1 ± 0.1 | 0.131^1^ |
| All data are expressed as mean ± SD  Abbreviations: CV, coefficient of variation; GMI, Glucose Monitoring Indicator; GRI, Glycemia Risk Index; SD, standard deviation; TAR, Time Above Range; TBR, Time Below Range; TIR, Time In Range; TTR, Tight Time in Range. | | | | |

**Supplementary Table 9 –** Change in CGM-related metrics from Time 0 to Time 1 in participants with diabetes duration ≥15 years

| **Diabetes duration ≥15 years (n=13)** | | | | |
| --- | --- | --- | --- | --- |
| **Variables** | **Time 0** | **Time 1** | **Difference** | **P** |
| TIR, % | 56.0 ± 18.3 | 68.0 ± 8.5 | 12.1 ± 11.7 | 0.003^1^ |
| TTR, % | 39.1 ± 10.7 | 43.9 ± 9.8 | 4.8 ± 12.5 | 0.186^1^ |
| TAR level 1, % | 27.2 ± 6.8 | 21.4 ± 3.5 | -5.8 ± 6.1 | 0.005^1^ |
| TAR level 2, % | 14.2 ± 13.6 | 9.4 ± 8.4 | -4.8 ± 7.8 | 0.045^1^ |
| TBR level 1, % | 2.2 ± 1.6 | 0.9 ± 0.8 | -1.3 ± 1.2 | 0.002^2^ |
| TBR level 2, % | 0.6 ± 0.7 | 0.2 ± 0.4 | -0.4 ± 0.7 | 0.054^2^ |
| CV, % | 35.9 ± 4.8 | 33.8 ± 5.8 | -2.1 ± 5.8 | 0.213^1^ |
| GMI, % | 7.5 ± 0.8 | 7.2 ± 0.4 | -0.3 ± 0.5 | 0.074^2^ |
| Mean glucose, mg/dl | 174.2 ± 32.5 | 163.4 ± 15.5 | -10.8 ± 21.5 | 0.094^1^ |
| SD, mg/dl | 60.6 ± 11.5 | 55.5 ± 12.9 | -5.1 ± 8.3 | 0.244^2^ |
| GRI | 51.6 ± 25.2 | 35.0 ± 13.7 | -16.5 ± 15.3 | 0.002^1^ |
| Total daily insulin dose, UI/die | 46.2 ± 15.9 | 45.1 ± 13.6 | -1.1 ± 10.0 | 0.692^1^ |
| Basal daily insulin dose, UI/die | 23.6 ± 9.8 | 23.6 ± 7.8 | -0.0 ± 5.1 | 0.979^1^ |
| Bolus daily insulin dose, UI/die | 22.6 ± 8.1 | 20.8 ± 9.5 | -1.8 ± 6.6 | 0.352^1^ |
| Insulin dose per kg, UI/kg/die | 0.6 ± 0.1 | 0.6 ± 0.1 | 0.0 ± 0.1 | 0.794^1^ |
| All data are expressed as mean ± SD  Abbreviations: CV, coefficient of variation; GMI, Glucose Monitoring Indicator; GRI, Glycemia Risk Index; SD, standard deviation; TAR, Time Above Range; TBR, Time Below Range; TIR, Time In Range; TTR, Tight Time in Range. | | | | |

**Supplementary Table 10 –** Change in CGM-related metrics from Time 0 to Time 1 in participants with diabetes duration <15 years.

| **Diabetes duration <15 years (n=7)** | | | | |
| --- | --- | --- | --- | --- |
| **Variables** | **Time 0** | **Time 1** | **Difference** | **P** |
| TIR, % | 59.7 ± 12.5 | 65.9 ± 8.5 | 6.1 ± 10.7 | 0.181^2^ |
| TTR, % | 34.6 ± 11.2 | 38.6 ± 4.5 | 4.0 ± 8.2 | 0.247^1^ |
| TAR level 1, % | 25.9 ± 6.9 | 23.2 ± 4.6 | -2.6 ± 5.9 | 0.294^1^ |
| TAR level 2, % | 11.9 ± 6.0 | 8.9 ± 4.9 | -3.0 ± 6.5 | 0.270^2^ |
| TBR level 1, % | 1.9 ± 0.7 | 1.4 ± 0.5 | -0.4 ± 0.8 | 0.200^2^ |
| TBR level 2, % | 1.0 ± 1.4 | 0.7 ± 1.1 | -0.3 ± 0.5 | 0.172^2^ |
| CV, % | 37.9 ± 1.6 | 35.6 ± 3.1 | -2.3 ± 3.6 | 0.137^1^ |
| GMI, % | 7.2 ± 0.5 | 7.2 ± 0.3 | -0.0 ± 0.5 | 0.895^2^ |
| Mean glucose, mg/dl | 168.7 ± 19.4 | 163.1 ± 11.8 | -5.6 ± 17.6 | 0.434^1^ |
| SD, mg/dl | 60.9 ± 14.6 | 58.3 ± 8.7 | -2.6 ± 10.3 | 0.532^1^ |
| GRI | 47.1 ± 16.2 | 38.4 ± 9.8 | -8.7 ± 14.2 | 0.155^2^ |
| Total daily insulin dose, UI/die | 45.7 ± 16.9 | 44.3 ± 15.1 | -1.4 ± 10.2 | 0.735^1^ |
| Basal daily insulin dose, UI/die | 22.1 ± 9.0 | 22.5 ± 7.7 | -0.4 ± 2.9 | 0.731^1^ |
| Bolus daily insulin dose, UI/die | 24.5 ± 6.8 | 21.2 ± 9.3 | -3.3 ± 7.8 | 0.308^1^ |
| Insulin dose per kg, UI/kg/die | 0.7 ± 0.2 | 0.7 ± 0.2 | 0.0 ± 0.1 | 0.840^1^ |
| All data are expressed as mean ± SD  Abbreviations: CV, coefficient of variation; GMI, Glucose Monitoring Indicator; GRI, Glycemia Risk Index; SD, standard deviation; TAR, Time Above Range; TBR, Time Below Range; TIR, Time In Range; TTR, Tight Time in Range. | | | | |

**Supplementary Figure 2 –** Percentage of subjects with TAR level 1 < 25% at Time 0 and Time 1 (P=0.112). TAR, Time Above Range.

**Supplementary Figure 3 –** Percentage of subjects with TAR level 2 < 5% at Time 0 and Time 1 (P=0.999). TAR, Time Above Range.

**Supplementary Figure 4 –** Percentage of subjects with TBR level 1 < 4% at Time 0 and Time 1 (P=0.231). TBR, Time Below Range.

**Supplementary Figure 5 –** Percentage of subjects with TBR level 2 < 1% at Time 0 and Time 1 (P=0.999). TBR, Time Below Range.

**Supplementary Figure 6 –** Distribution of participants across GRI zones from Time 0 to Time 1. GRI value: zone A: 0-20; zone B: 20-40; zone C: 40-60; zone D: 60-80; zone E: 80-100.

Zone A (P=0.999), zone B (P=0.057), zone C (P=0.747), zone D (P=0.605), zone E (P=0.231).

**STROBE Statement —** Checklist of items that should be included in reports of observational studies.

|  | **Item No** | **Recommendation** | **Pag** |
| --- | --- | --- | --- |
| **Title and abstract** | 1 | (*a*) Indicate the study’s design with a commonly used term in the title or the abstract | 1, title page |
|  |  | (*b*) Provide in the abstract an informative and balanced summary of what was done and what was found | 2, abstract |
| **Introduction** | | |  |
| Background/rationale | 2 | Explain the scientific background and rationale for the investigation being reported | 3,4, introduction |
| Objectives | 3 | State specific objectives, including any prespecified hypotheses | 3,4, introduction |
| **Methods** | | |  |
| Study design | 4 | Present key elements of study design early in the paper | 4, study design and population |
| Setting | 5 | Describe the setting, locations, and relevant dates, including periods of recruitment, exposure, follow-up, and data collection | 4, study design and population |
| Participants | 6 | (*a*) *Cohort study*—Give the eligibility criteria, and the sources and methods of selection of participants. Describe methods of follow-up  *Case-control study*—Give the eligibility criteria, and the sources and methods of case ascertainment and control selection. Give the rationale for the choice of cases and controls  *Cross-sectional study*—Give the eligibility criteria, and the sources and methods of selection of participants | 4, study population |
|  |  | (*b*) *Cohort study*—For matched studies, give matching criteria and number of exposed and unexposed  *Case-control study*—For matched studies, give matching criteria and the number of controls per case |  |
| Variables | 7 | Clearly define all outcomes, exposures, predictors, potential confounders, and effect modifiers. Give diagnostic criteria, if applicable | 5,6, statistical analysis |
| Data sources/ measurement | 8* | For each variable of interest, give sources of data and details of methods of assessment (measurement). Describe comparability of assessment methods if there is more than one group | 5,6, materials and methods |
| Bias | 9 | Describe any efforts to address potential sources of bias | 5,6, statistical analysis |
| Study size | 10 | Explain how the study size was arrived at | 4, study population |
| Quantitative variables | 11 | Explain how quantitative variables were handled in the analyses. If applicable, describe which groupings were chosen and why | 5,6, statistical analysis |
| Statistical methods | 12 | (*a*) Describe all statistical methods, including those used to control for confounding | 5,6, statistical analysis |
|  |  | (*b*) Describe any methods used to examine subgroups and interactions | 5,6, statistical analysis |
|  |  | (*c*) Explain how missing data were addressed | 4,5,6, materials and methods |
|  |  | (*d*) *Cohort study*—If applicable, explain how loss to follow-up was addressed  *Case-control study*—If applicable, explain how matching of cases and controls was addressed  *Cross-sectional study*—If applicable, describe analytical methods taking account of sampling strategy |  |
|  |  | (*e*) Describe any sensitivity analyses |  |

| **Results** | | |  |
| --- | --- | --- | --- |
| Participants | 13* | (a) Report numbers of individuals at each stage of study—eg numbers potentially eligible, examined for eligibility, confirmed eligible, included in the study, completing follow-up, and analysed | 6,7 results |
|  |  | (b) Give reasons for non-participation at each stage | 6 results |
|  |  | (c) Consider use of a flow diagram | Supplementary Figure 1 |
| Descriptive data | 14* | (a) Give characteristics of study participants (eg demographic, clinical, social) and information on exposures and potential confounders | 6,7, results |
|  |  | (b) Indicate number of participants with missing data for each variable of interest | 6,7, results |
|  |  | (c) *Cohort study*—Summarise follow-up time (eg, average and total amount) | 6,7, results |
| Outcome data | 15* | *Cohort study*—Report numbers of outcome events or summary measures over time | 6,7, results |
|  |  | *Case-control study—*Report numbers in each exposure category, or summary measures of exposure |  |
|  |  | *Cross-sectional study—*Report numbers of outcome events or summary measures |  |
| Main results | 16 | (*a*) Give unadjusted estimates and, if applicable, confounder-adjusted estimates and their precision (eg, 95% confidence interval). Make clear which confounders were adjusted for and why they were included | 6,7, results |
|  |  | (*b*) Report category boundaries when continuous variables were categorized | 5,6, results |
|  |  | (*c*) If relevant, consider translating estimates of relative risk into absolute risk for a meaningful time period |  |
| Other analyses | 17 | Report other analyses done—eg analyses of subgroups and interactions, and sensitivity analyses | 6,7, results |
| **Discussion** | | |  |
| Key results | 18 | Summarise key results with reference to study objectives | 7,8,9, discussion |
| Limitations | 19 | Discuss limitations of the study, taking into account sources of potential bias or imprecision. Discuss both direction and magnitude of any potential bias | 7,8,9, discussion |
| Interpretation | 20 | Give a cautious overall interpretation of results considering objectives, limitations, multiplicity of analyses, results from similar studies, and other relevant evidence | 7,8,9, discussion |
| Generalisability | 21 | Discuss the generalisability (external validity) of the study results | 10, discussion |
| **Other information** | | |  |
| Funding | 22 | Give the source of funding and the role of the funders for the present study and, if applicable, for the original study on which the present article is based |  |
